# *RBMS3* at 3p24 Inhibits Nasopharyngeal Carcinoma Development via Inhibiting Cell Proliferation, Angiogenesis, and Inducing Apoptosis

**DOI:** 10.1371/journal.pone.0044636

**Published:** 2012-09-05

**Authors:** Juan Chen, Dora Lai-Wan Kwong, Cai-Lei Zhu, Lei-Lei Chen, Sui-Sui Dong, Li-Yi Zhang, Jun Tian, Chu-Bo Qi, Ting-Ting Cao, Alissa Michelle Go Wong, Kar-Lok Kong, Yan Li, Ming Liu, Li Fu, Xin-Yuan Guan

**Affiliations:** 1 Department of Clinical Oncology, The University of Hong Kong, Pokfulam, Hong Kong, China; 2 Cancer Center, Union Hospital, Tongji Medical College, Huazhong University of Science and Technology, Wuhan, China; 3 State Key Laboratory of Oncology in Southern China, Cancer Center, Sun Yat-Sen University, Guangzhou, China; 4 Department of Biochemistry and Molecular Biology, Tongji Medical College, Huazhong University of Science and Technology, Wuhan, China; 5 Department of Pathology, Hubei Cancer Hospital, China; The Chinese University of Hong Kong, Hong Kong

## Abstract

Deletion of the short arm of chromosome 3 is one of the most frequent genetic alterations in many solid tumors including nasopharyngeal carcinoma (NPC), suggesting the existence of one or more tumor suppressor genes (TSGs) within the frequently deleted region. A putative TSG *RBMS3* (RNA binding motif, single stranded interacting protein 3), located at 3p24-p23, has been identified in our previous study. Here, we reported that downregulation of *RBMS3* was detected in 3/3 NPC cell lines and 13/15 (86.7%) primary NPC tissues. Functional studies using both overexpression and suppression systems demonstrated that RBMS3 has a strong tumor suppressive role in NPC. The tumor suppressive mechanism of *RBMS3* was associated with its role in cell cycle arrest at the G1/S checkpoint by upregulating p53 and p21, downregulating cyclin E and CDK2, and the subsequent inhibition of Rb-ser780. Further analysis demonstrated that RBMS3 had a pro-apoptotic role in a mitochondrial-dependent manner via activation of caspase-9 and PARP. Finally, RBMS3 inhibited microvessel formation, which may be mediated by down-regulation of MMP2 and β-catenin and inactivation of its downstream targets, including cyclin-D1, c-Myc, MMP7, and MMP9. Taken together, our findings define a function for *RBMS3* as an important tumor suppressor gene in NPC.

## Introduction

Nasopharyngeal carcinoma (NPC) is a distinct and geographically important disease [1], which accounts for 80,000 new cases and 50,000 deaths per year [2]. The majority (75–90%) of newly diagnosed NPC patients have loco-regionally advanced disease, commonly with cervical nodal metastases [3]. Currently, the standard of care for these patients consists of concurrent chemo-radiotherapy with cisplatin-based regimens, generally followed by adjuvant chemotherapy. The cause for NPC development is complex, including viral, genetic and environmental factors[4]–[6]. It is widely accepted that infection by the Epstein-Barr virus (EBV) plays a vital role in the pathogenesis of NPC; however, the molecular pathogenesis is also associated with the inactivation of tumor suppressor genes (TSGs). To date, the exact cellular and molecular mechanisms leading to NPC have not been systematically evaluated.

The 3p chromosomal region is frequently deleted in multiple solid tumors [9], suggesting the existence of one or more TSGs contributing to the risk of developing NPC. Through massive expression profiling and epigenetic characterization, we and others have identified several interesting 3p targets genes in human cancers, including *BLU* [10], *RBMS3* [11] and two closely located 3p22 genes, *DLEC1* [12] and *PLCD1* [13], [14]. *RBMS3*, a gene located at 3p24-p23, encodes an RNA-binding protein that belongs to the c-Myc gene single-strand binding protein (MSSPs) family [15]. The MSSP family members are single strand DNA-binding proteins that cooperate with the Myc protein to regulate DNA replication, gene transcription, cell cycle progression, and induction of apoptosis. C-Myc is over-expressed in most cancers and executes its diverse functions through transcriptional regulation of its target genes [16]. Over-expression of c-Myc was also found as a frequent genetic abnormality in NPC [17], [18]. The RBMS3 protein was isolated through its binding to an upstream element of the alpha2 (I) collagen promoter [19]. The RBMS3 protein localizes in the cytoplasm, suggesting it may be involved in a cytoplasmic function such as controlling RNA metabolism, rather than transcription. Multiple alternatively spliced transcript variants encoding various isoforms of *RBMS3* have been found. Although *RBMS3* could be a potential cooperator of the Myc protein, its role in the pathogenesis of NPC remains unclear. In the present study, the expression pattern of RBMS3 in primary NPCs and NPC cell lines was investigated. The tumor suppressive effects and corresponding mechanisms of RBMS3 were characterized.

## Results

### RBMS3 is Frequently Down-regulated in NPC

**Figure 1:**
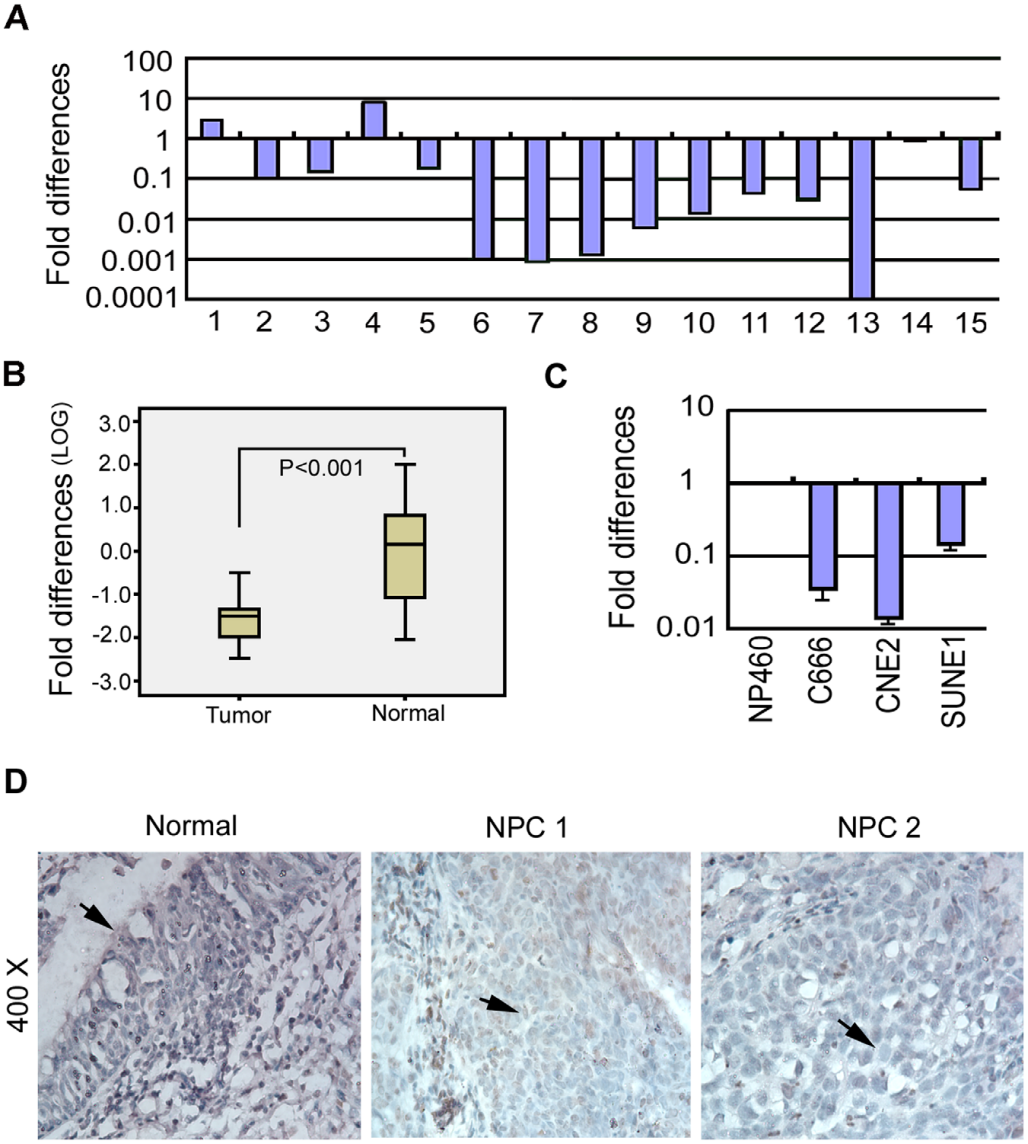
Downregulation of RBMS3 in nasopharyngeal carcinoma (NPC). (**A**) Expression of *RBMS3* in 15 primary NPC cases was compared using qPCR between tumor tissues (T) and their paired normal tissue (N). GAPDH was set as an internal control. (**B**) *RBMS3* expression was normalized by internal control *GAPDH*. Statistical analysis confirmed the qPCR results (p<0.001). (**C**) qPCR analysis of *RBMS3* expression in three NPC cell lines (C666, CNE2 and SUNE1). The fold changes of *RBMS3* expression were compared with the immortalized NP cell line, NP460. (**D**) Immunohistochemical detection of RBMS3 protein in NPC tissue samples and non-tumor nasopharyngeal tissues. Normal: strong positive staining for RBMS3 protein in normal nasopharyngeal epitheliums (arrowhead). NPC1: weak positive staining for RBMS3 protein in NPC tissues (arrowhead). NPC2: negative staining for RBMS3 protein in NPC tissues (arrowhead).

Quantitative real-time PCR (qRT-PCR) was performed to evaluate the expression levels of *RBMS3* in 15 pairs of primary NPCs and their corresponding non-tumor samples. Down-regulation of *RBMS3* was detected in 13/15 (86.7%) NPC tissues compared to their normal counterparts (Fig. 1A). Furthermore, the box plot showed a highly significant difference in the mean expression levels of *RBMS3* between NPC tumors and non-tumor samples (p<0.001; Fig. 1B). We next examined *RBMS3* expression in NPC cell lines. The result showed that *RBMS3* was downregulated in all three tested NPC cell lines (C666, CNE2 and SUNE1) compared to the immortalized nasopharyngeal (NP) cell line, NP460 (Fig. 1C). The protein expression level of RBMS3 was also evaluated in 30 pairs of primary NPCs and non-tumor samples by immunohistochemical staining (IHC). Moderate or strong nuclear staining of RBMS3 was detected in 30 non-tumor tissues, whereas no or weak nuclear staining of RBMS3 was observed in 24/30 (80.0%) of NPC tumor tissues (Fig. 1D).

### RBMS3 has Tumor Suppressive Ability

**Figure 2:**
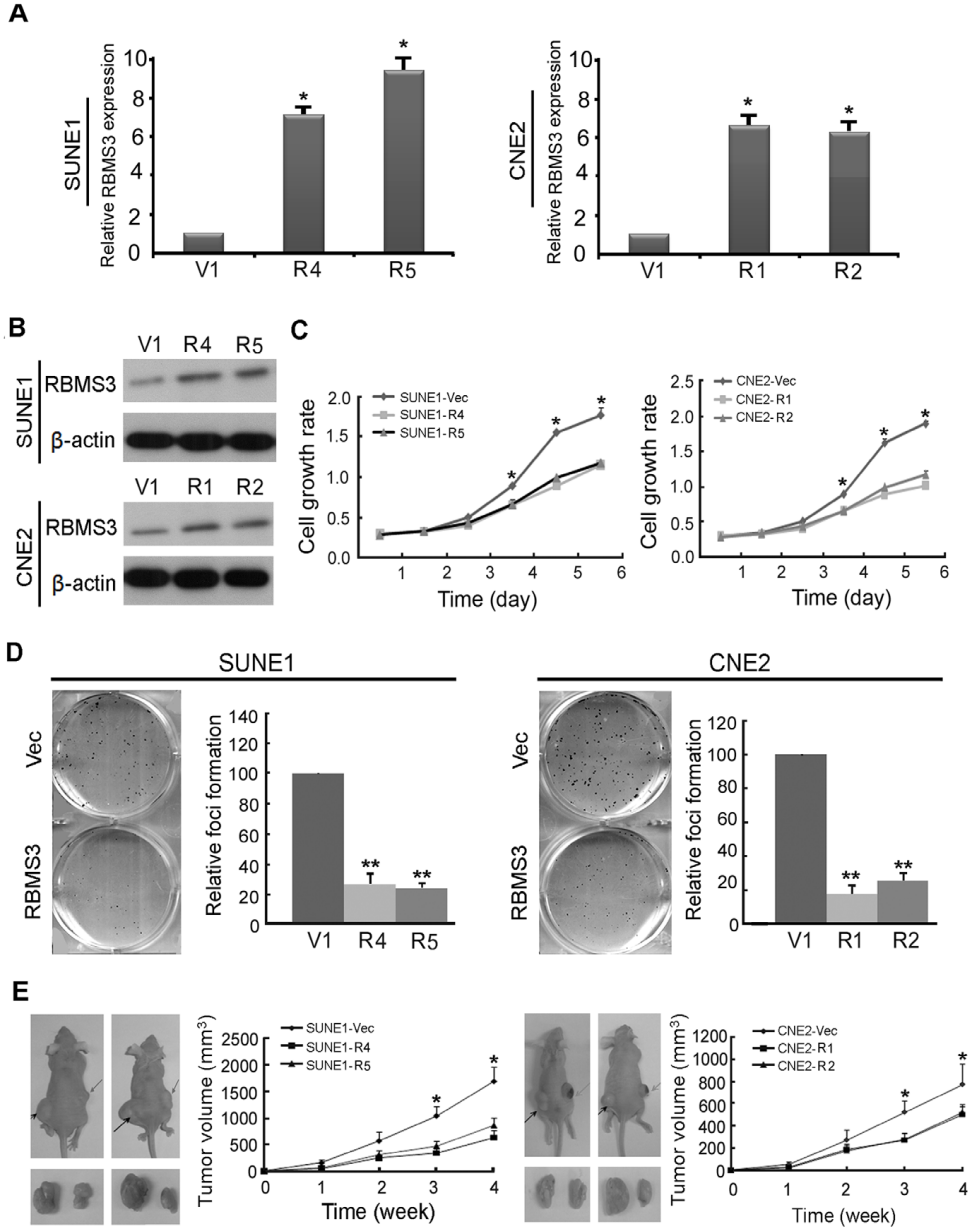
Tumor suppressor function of *RBMS3* in NPC cells. Expression of *RBMS3* in *RBMS3*-transfected SUNE1 (SUNE1-R4 and R5) and CNE2 cells (CNE2-R1 and R2) was confirmed by quantitative PCR (**A**) and Western blot (**B**). Empty vector-transfected cells (SUNE1-V1, CNE2-V1) were used as control. (**C**) An MTT assay was used to compare cell growth rate between *RBMS3*- and empty vector-transfected NPC cells. The results found that cell growth rate was significantly decreased in *RBMS3*-transfected SUNE1 and CNE2 cells (* p<0.05; ** p<0.001, Student’s *t*-test). Values were expressed as mean ± SD of three independent experiments. (**D**) Foci formation assay showed that the number of foci was significantly decreased in *RBMS3*-transfected SUNE1 and CNE2 cells compared to the control cells, respectively (** p<0.001, Student’s *t*-test). The results were expressed as mean ± SD of three independent experiments. (**E**) Representatives of tumor formation in nude mice. Tumors induced by SUNE1-V1 (*left*) and SUEN1-RBMS3 (*right*) were indicated by arrows, respectively. Excised tumors were shown in the bottom. Summary of tumor growth rates in nude mice induced by *RBMS3*- and empty vector-transfected NPC cells. The average tumor volume was expressed as mean ± SD in 10 inoculated sites for each group (* p<0.05).

To investigate whether *RBMS3* has tumor suppressive ability, *RBMS3* was stably transfected into 2 NPC cell lines (SUNE1 and CNE2), and 4 clones (SUNE1-R4, SUNE1-R5, CNE2-R1 and CNE2-R2) were selected for functional studies. Empty vector-transfected cells were used as control (SUNE1-V1 and CNE2-V1). Expression of *RBMS3* in SUNE1-R4, SUNE1-R5, CNE2-R1 and CNE2-R2 cells was confirmed by qPCR (Fig. 2A) and Western blot analysis (Fig. 2B). Tumor suppressive function of *RBMS3* was studied by cell growth assay, foci formation assay, and tumor xenograft experiment. Cell growth assay showed that the growth rates were significantly decreased in SUNE1-R4, SUNE1-R5, CNE2-R1 and CNE2-R2 cells (p<0.05, Student’s *t*-test) compared to SUNE1-V1 cells and CNE2-V1 cells, respectively (Fig. 2B). Focus formation assay showed that *RBMS3* could significantly inhibit foci formation (p<0.001, Student’s *t*-test) in SUNE1-R4, SUNE1-R5, CNE2-R1 and CNE2-R2 cells compared to control cells (Fig. 2C).

The tumor suppressive potential of *RBMS3* was also evaluated by xenograft tumor formation in athymic nude mice. Subcutaneous tumor formation was observed in all nude mice injected with SUNE1-V1 (n = 10) and CNE2-V1 (n = 10) cells 28 days post-injection. Xenograft tumor growth curve showed that tumors induced by SUNE1-R4 and SUNE1-R5 cells grew significantly slower than the SUNE1-V1 cells (p<0.05) (Fig. 2D). The average volume of the tumors induced by SUNE1-R4 (630.00±135.18 mm^3^) and SUNE1-R5 (864.00±144.68 mm^3^) cells were significantly smaller compared to the tumors induced by SUNE1-V1 cells (1687.80±270.37 mm^3^, p<0.05) (Fig. 2D). Similarly, the average volume of the tumors induced by CNE2-R1 (501.98±73.12 mm^3^) and CNE2-R2 (522.13±74.19 mm^3^) were significantly reduced compared to the tumors induced by CNE2-V1 cells (770.46±187.07 mm^3^, p<0.05) (Fig. 2D).

### RBMS3 Arrests Cell Cycle at G1-S Checkpoint

**Figure 3:**
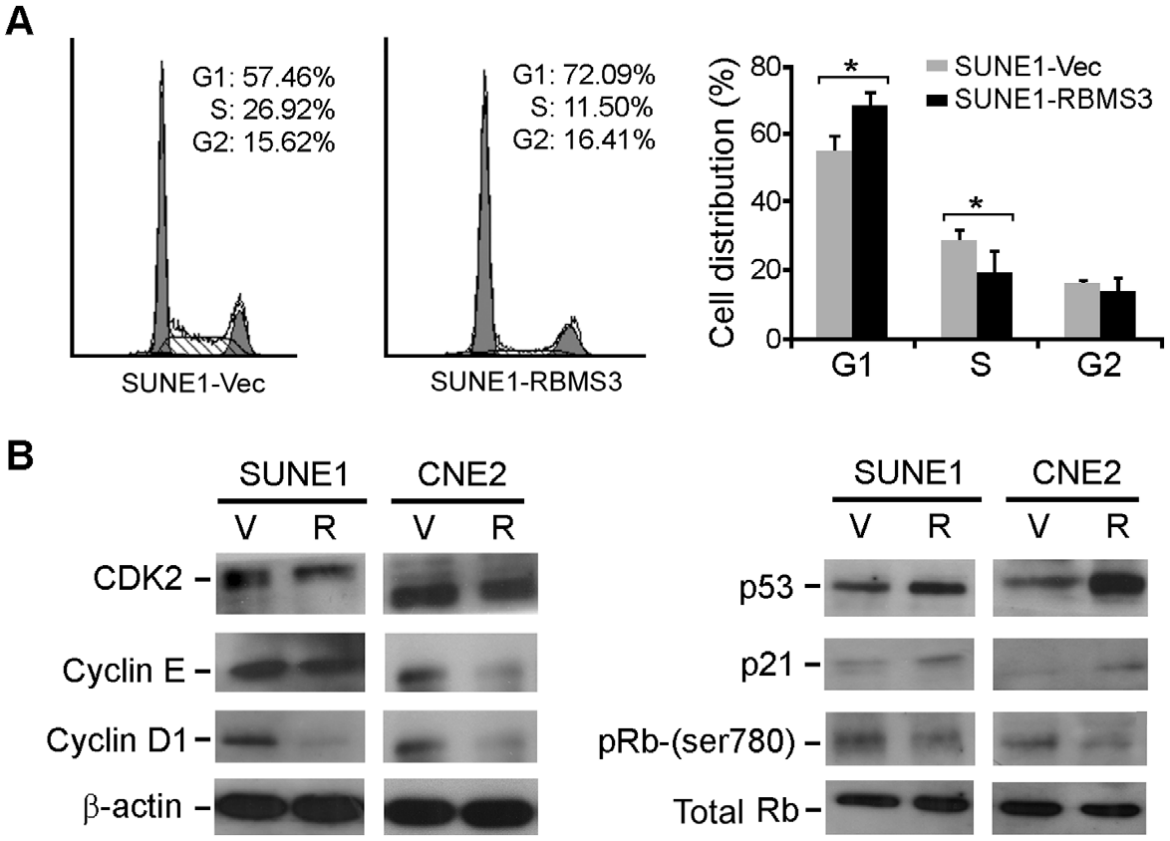
*RBMS3* arrests cell cycle at the G1/S checkpoint. (**A**) Representative and summary of DNA content detected by flow cytometry showed that the percentage of cells in the S phase was lower while the percentage of cells in the G1 phase was higher in SUNE1-RBMS3 cells than that in SUNE1-V1 cells. (* p<0.05, Student’s *t*-test). Values were expressed as mean ± SD of three independent experiments. (**B**) Protein expressions of cyclin D1, cyclin E, CDK2, p53, p21, Rb and Rb (Ser780) were compared between *RBMS3*- and empty vector-transfected NPC cells. β-actin was used as a loading control.

To understand the tumor suppressive mechanism of *RBMS3*, flow cytometry was performed to compare the DNA content between the SUNE1-Vec and SUNE1-RBMS3 cells. The results showed that the proportions of SUNE1-RBMS3 cells in the G1-phase and S-phase were significantly increased and decreased, respectively, in the SUNE1-RBMS3 cells (p<0.05) compared with SUNE1-Vec cells, suggesting that *RBMS3* was able to arrest cell cycle at G1/S checkpoint (Fig. 3A). Western blot analysis found that the G1/S checkpoint promoting factors (CDK2, cyclin E and cyclin D1) and inhibiting factors (p53 and p21) were downregulated and upregulated in *RBMS3*-tansfected NPC cells, respectively, compared to control cells (Fig. 3B). Since CDK2 plays a critical role in the inactivation of Rb, the level of the inactive form (phosphorylated) of Rb was also compared between *RBMS3*-transfected and vector tarnsfected NPC cells by western blotting. The result showed that inactive form of Rb (Ser780) was reduced in *RBMS3*-transfected cells compared to control cells. However, the total amount of Rb protein did not change significantly (Fig. 3B).

### Ectopic Expression of RBMS3 Induces Apoptosis

**Figure 4:**
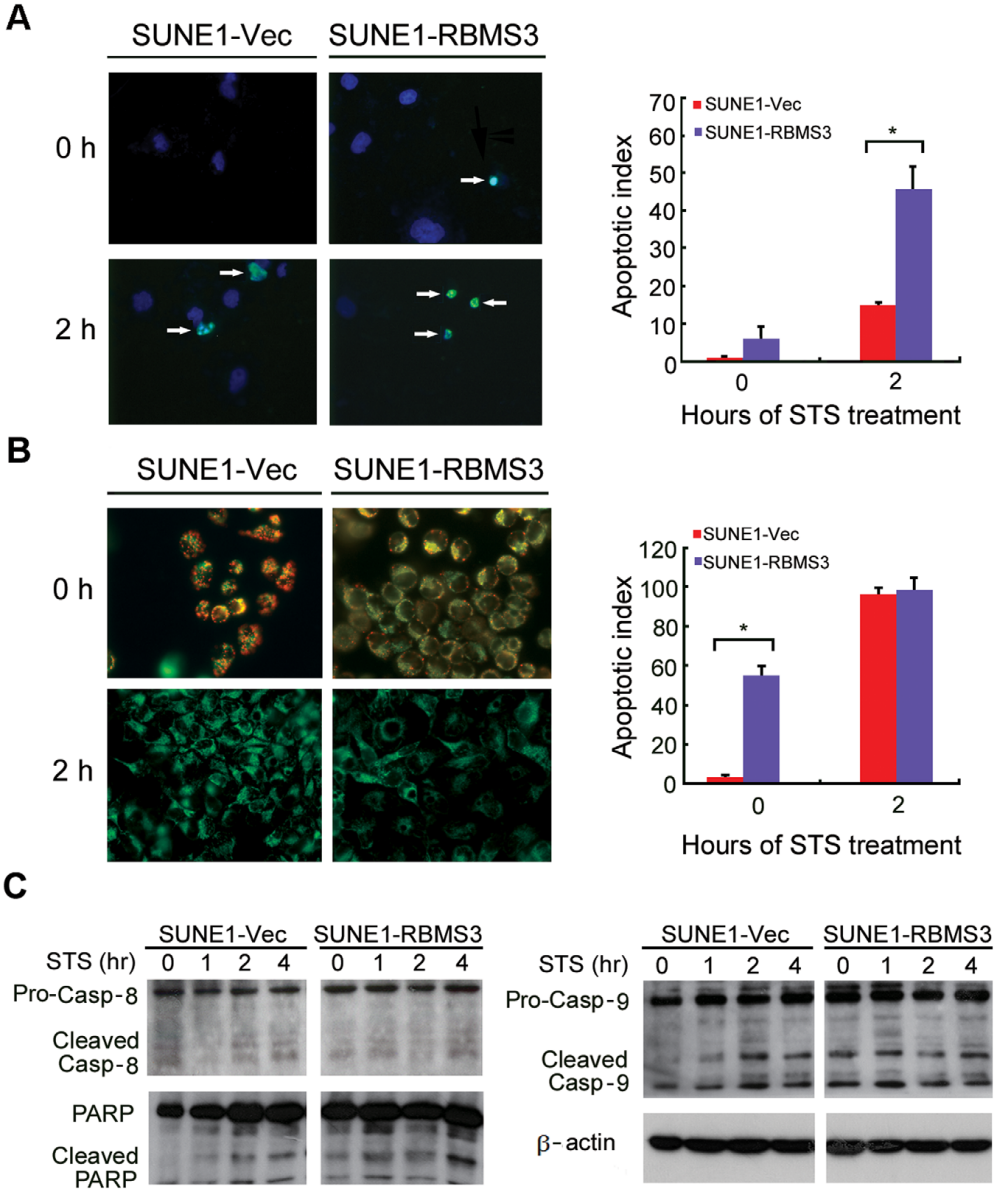
Ectopic expression of *RBMS3* induces apoptosis. (**A**) Representative images of TUNEL staining. After STS treatment, more apoptotic cells (green) were detected in SUNE1-RBMS3 cells compared to SUNE1-V1 cells (arrowhead). The apoptotic indexes of SUNE1-RBMS3 cells and SUNE1-V1 cells (before and after STS treatment) were summarized in the right panel (*p<0.05). (**B**) This event was measured by loss of mitochondrial ΔΨm using JC-1 dye. Red or orange fluorescence indicates intact mitochondria, whereas green fluorescence indicated a collapse in mitochondrial ΔΨm. Prior to STS treatment the SUNE1-RBMS3 cells reveal a significant ΔΨ_m_ loss (green fluorescence, ×400) and higher apoptotic index compared to SUNE1-V1 cells (red/orange fluorescence; left panel) (*p<0.05). (**C**) The cleavages of caspase-9, caspase-8, and PARP were compared between *RBMS3*-transfected and empty vector-transfected SUNE1 cells. β-actin was used as loading control.

To explore whether *RBMS3* has a pro-apoptotic effect, the apoptotic index was compared between SUNE1-Vec and SUNE1-RBMS3 cells by TUNEL staining. Prior to straurosporine (STS) treatment, TUNEL analysis revealed that the apoptotic index in SUNE1-Vec cells (0.5%±0.9%) was lower than SUNE1-RBMS3 cells (7.1%±6.0%; p = 0.057). After STS treatment, the apoptotic index in SUNE1-Vec (14.8%±4.1%) was significantly lower than that of SUNE1-RBMS3 (45.3%±4.5%; p<0.05), confirming that *RBMS3* had a pro-apoptotic ability (Fig. 4A). To elucidate the molecular basis of apoptosis, we examined the potential for pro-apoptotic mitochondrial permeability transition by measuring the loss of mitochondrial ΔΨm using JC-1 dye. Red or orange fluorescence indicates intact mitochondria, whereas green fluorescence indicates a collapse in mitochondrial ΔΨm. The results showed that the mitochondrial permeability and apoptotic index in RBMS3-transfected cells were significantly higher than those of control cells even prior to STS treatment (Fig. 4B). Western blot analysis also indicated that the cleavage of caspase-9 and PARP was dramatically increased in SUNE1-RBMS3, however, no change was observed for caspase-8 (Fig. 4C).

### RBMS3 Inhibits Angiogenesis

**Figure 5:**
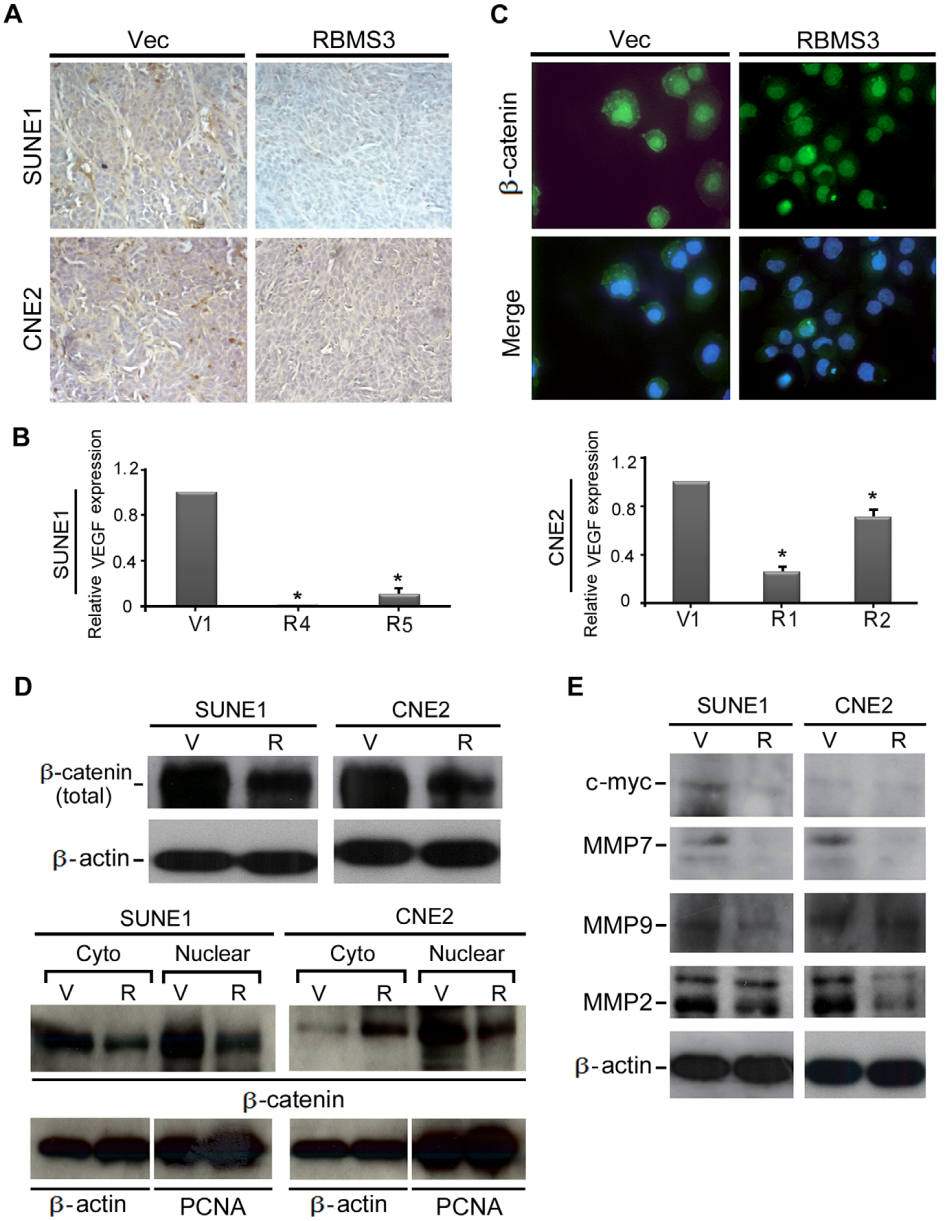
*RBMS3* inhibits angiogenesis. (**A**) The tumor sections were stained for the endothelial cell marker CD34 (×200). The RBMS3-induced tumors had a lower degree of microvessel densities compared to empty vector-induced tumors. (**B**) Expression of *VEGF* in *RBMS3*-transfected SUNE1 and CNE2 cells was confirmed by qPCR. Empty vector-transfected cells were used as control. (**C**) Representative IF images showed that β-catenin was mainly localized in the nucleus of SUNE1-RBMS3 cells, whereas it was mainly localized in cell membrane and cytoplasm in SUNE1-V1 cells. (**D**) Western blot analysis of β-catenin from whole cell, nuclear and cytosolic extract was compared between in *RBMS3*- and empty vector-transfected SUNE1 and CNE2 cells. β-actin and PCNA were used as loading control. (**E**)Western blot analysis of c-Myc, MMP7, MMP9 and MMP2 was compared between *RBMS3*- and empty vector-transfected SUNE1 and CNE2 cells. β-actin was used as loading control.

To study the potential effect of RBMS3 on angiogenensis, the development of microvessel in tumor sections of mouse xenografts was examined by IHC staining with a vascular endothelial cell marker CD34. As shown in Fig. 5A, a robust angiogenic response, as determined by high CD34 positivity, was observed in the empty vector-induced tumors. Conversely, CD34-positive vessels were rarely found within the RBMS3-induced tumors. The number of vessels counted in RBMS3-induced and empty vector-induced tumors was summarized in Table 1. Furthermore, the mRNA expression level of VEGF was also compared between *RBMS3*-transfected and vector-transfected NPC cells by qPCR analysis. As shown in Fig. 5B, the expression of VEGF was dramatically decreased in RBMS3-transfected NPC cells compared to control cells. (p<0.05, Fig. 5B).

**Table 1:** Quantitation of microvessels formed in empty vector and RBMS3-transfected NPC cells induced mouse tumor xenografts (n = 10 tumors/group).

Cell lines		aVessel count		*P*
		Vec	RBMS3	
SUNE1		78.6±16.9	23.4±8.5	<0.001**
CNE2		87.9±27.1	29.6±6.7	<0.001**

a Values represent mean±standard error.

** Statistically significant as compared to control cells.

A recent study found that silencing β-catenin expression by RNA interference could inhibit angiogenesis-related gene expression (e.g., MMP9, MMP2, and VEGF) in hepatocellular carcinoma cells [20]. We next studied whether RBMS3 could intercept the expression of β-catenin in NPC cells. As shown in Fig. 5C and Fig. 5D, β-catenin from both whole cell extracts and nuclear fractionation was downregulated in *RBMS3*-transfected NPC cells compared to control cells. The downstream targets of β-catenin including C-Myc, MMP7, MMP9, and MMP2 (the latter two are angiogenesis-related proteins) were also detected in RBMS3-transfected NPC cells. As expected, all the β-catenin downstream targets including the two angiogenesis-related proteins MMP9 and MMP2 were significantly downregulated in RBMS3 transfected NPC cells, suggesting that RBMS3 has a strong angiogenesis inhibiting role in NPC (Fig. 5E).

**Figure 6:**
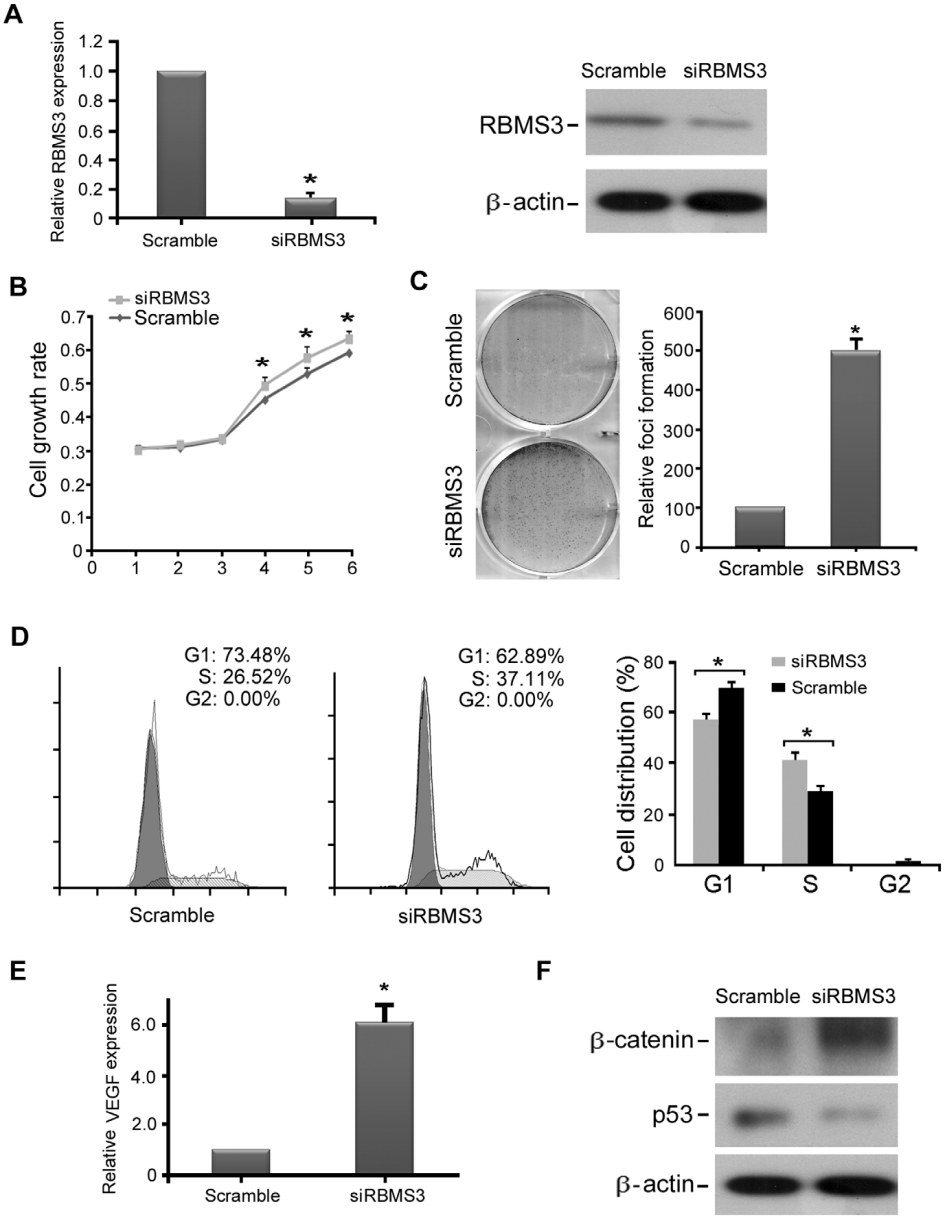
*RBMS3*-silenced NP460 cell showed malignant features. (**A**) siRNA against *RBMS3* effectively reduced the mRNA and protein expression of RBMS3 in NP460 cells compared to siScramble-transfected cells. (**B**) An MTT assay was used to compare cell growth rates between siRBMS3- and siScramble-transfected NP460 cells (* p<0.05; ** p<0.001, Student’s *t*-test). Values were expressed as mean ± SD of three independent experiments. (**C**) Foci formation assay showed that the number of foci was significantly increased in siRBMS3-transfected NP460 cells compared to control cells (* p<0.05, Student’s *t*-test). The results were expressed as mean ± SD of three independent experiments. (**D**) Representative and summary of DNA content detected by flow cytometry showed that the percentage of cells in the S phase was higher while the percentage of cells in the G1 phase was lower in siRBMS3-transfected NP460 cells than that in control cells. (* p<0.05, Student’s *t*-test). (**E**) Expression of *VEGF* in siRBMS3- and siScramble-transfected NP460 cells was confirmed by qPCR. (* p<0.05, Student’s *t*-test). The results were expressed as mean ± SD of three independent experiments. (**F**) β-catenin was significantly up-regulated while p53 was significantly down-regulated in siRBMS3-transfected NP460 cells compared to control cells by Western blot analysis. β-actin was used as a loading control.

To further demonstrate the tumor-suppressive function of RBMS3, RNAi was used to knockdown endogenous *RBMS3* expression in NP460 cells. The silencing effect was confirmed by both qPCR and western blotting (Fig. 6A). The function of *RBMS3* in RBMS3 knockdown NP460 cells was studied by cell growth assay, foci formation assay, and cell cycle analysis. The results showed that knockdown of RBMS3 in NP460 cells could increase cell growth rate (Fig. 6B), enhance foci formation ability (Fig. 6C), and promote cell cycle (Fig. 6D) compared to control cells. Moreover, we found that the expression of VEGF was significantly upregulated in RBMS3 knockdown NP460 cells by qPCR analysis (Fig. 6E), whereas β-catenin was upregulated and p53 was downregulated in RBMS3 knockdown NP460 cells by western blotting (Fig. 6F), as compared to control cells. These observations further support that RBMS3 is an important tumor suppressor with anti-proliferation and anti-angiogenesis abilities in NPC.

## Discussion

The etiology of NPC is complex, and includes viral, genetic and environmental factors [4]–[6]. The earliest and most common genetic change observed in NPC patients maps to the short arm of chromosome 3 where deletions are observed frequently. One candidate tumor suppressor gene, *RBMS3*, which resides on human chromosome 3p24-3p23, is widely expressed in the embryo as well as in the adult organisms. In the present study, downregulation of *RBMS3* was frequently detected in NPCs at both the mRNA and protein levels, suggesting that *RBMS3* might play a pivotal role in the NPC development and progression. The observation that this protein localizes in the nuclear suggests that it may be involved in a nuclear fuction such as controlling RNA transcription. The tumor-suppressive function of *RBMS3* was characterized in two NPC cell lines (SUNE1 and CNE2). Both *in vitro* (cell growth rate, colony formation in soft agar and foci formation) and *in vivo* (tumor formation in nude mouse) assays demonstrated that *RBMS3* had a strong tumor suppressive potential in NPC cells.

Molecular analysis found that the tumor-suppressive effect of *RBMS3* was closely associated with its role in cell cycle arrest at G1/S checkpoint via upregulation of p53 and p21, and downregulation of cyclin D1, cyclin E/CDK2 and Rb-ser780. The increased expression of p53, which is a DNA-binding protein and functions as a tumor suppressor, can induce the expression of p21. p21 has a pivotal role in the G1/S transition through inhibiting the cyclin E/CDK2 complex [21]. Activation of the cyclin E/CDK2 complex can destruct Rb-E2F binding, which contributes to the reduction of phosphorylation form of Rb, and finally activates the transcription of genes necessary for S-phase entry and cell cycle progression [22], [23]. These findings indicate that *RBMS3* could be necessary for an orderly G1/S transition in NPC cells.

Another data supporting that *RBMS3* is a tumor suppressor in NPC was its role in inducing apoptosis. Notably, the apoptotic index between RBMS3 transfected NPC cells and control cells showed significant difference after STS treatment, indicating a unique mechanism underlying the strong pro-apoptotic effect of RBMS3 in NPC cancer cells. It has been reported that RBMS3 increased the expression of Prx1 transcription factor [19], which is a tumor suppressor in esophageal squamous cell carcinoma [24]. Prx1, as a novel interaction partner for the lifespan regulator protein p66Shc, binds to the N-terminal domain of p66Shc [25], [26], and subsequently induces changes in the permeability transition of mitochondrial membranes and results in the release of cytochrome C and apoptosome activation [27], [28]. Interestingly, our results also found that the pro-apoptotic role of RBMS3 was mediated through the intrinsic mitochondrial pathway, as caspase-9 and mitochondrial permeability, but not caspase-8, was increased. However, whether RBMS3 could induce the expression of Prx1 and lead to the activation of a similar apoptotic pathway need to be further investigated.

Whether or not the growth of vessels occurs in a tissue depends on the equilibrium between the relative amounts of molecules that induce and inhibit angiogenesis [29]. A growing body of evidence illustrates that the inactivation of TSG and activation of oncogenes play an important role in this phenotypic switch. For instance, oncogenic stimuli such as ras, v-src and c-jun [30]–[33] and inactivation of p53, VHL, and RB2/P130 results in an increased VEGF production [30], [34], [35]. Our data demonstrate that RBMS3 can modulate the angiogeneic balance and inhibit the microvessel formation potentially through increasing the expression of p53 and decreasing the expression of MMP9 and MMP2 [36].

β-catenin is maintained at low levels in quiescent cells by interaction with protein kinases, such as adenomatous polyposis coli, casein kinase 1, and glycogen synthase kinase 3 [37]. The aberrant cytoplasmic accumulation of β-catenin induces Tcf/Lef-mediated transcriptional activity, and enhances the invasion and migration of oral squamous cell carcinoma [38]. The translocation of β-catenin into the nucleus can initiate carcinogenesis when Wnt is present [39]. β-catenin levels in nucleus are increased in 92% of NPC tumors, making β-catenin an important component in NPC development [40], [41]. Our data showed that β-catenin from both whole cell extracts and nuclear fractionation was significantly downregulated in *RBMS3* transfected NPC cells compared to control cells, suggesting that *RBMS3* could inhibit the nuclear translocation of β-catenin in NPC cells. The inhibition of β-catenin nuclear translocation in *RBMS3*-expressing cells was further confirmed by detecting downregulation of its targets including c-Myc, MMP7, MMP9 and MMP2. Overexpression of c-Myc has been detected in about 90% of NPCs which was associated with poor survival rate in NPC patients [42], [43]. MMP7 is one of the well-known targets of β-catenin. It has been reported that MMP7 plays an essential role in cancer invasion and metastases [44], [45]. In this study, MMP7 expression could be effectively downregulated when *RBMS3* was introduced into NPC cells.

**Figure 7:**
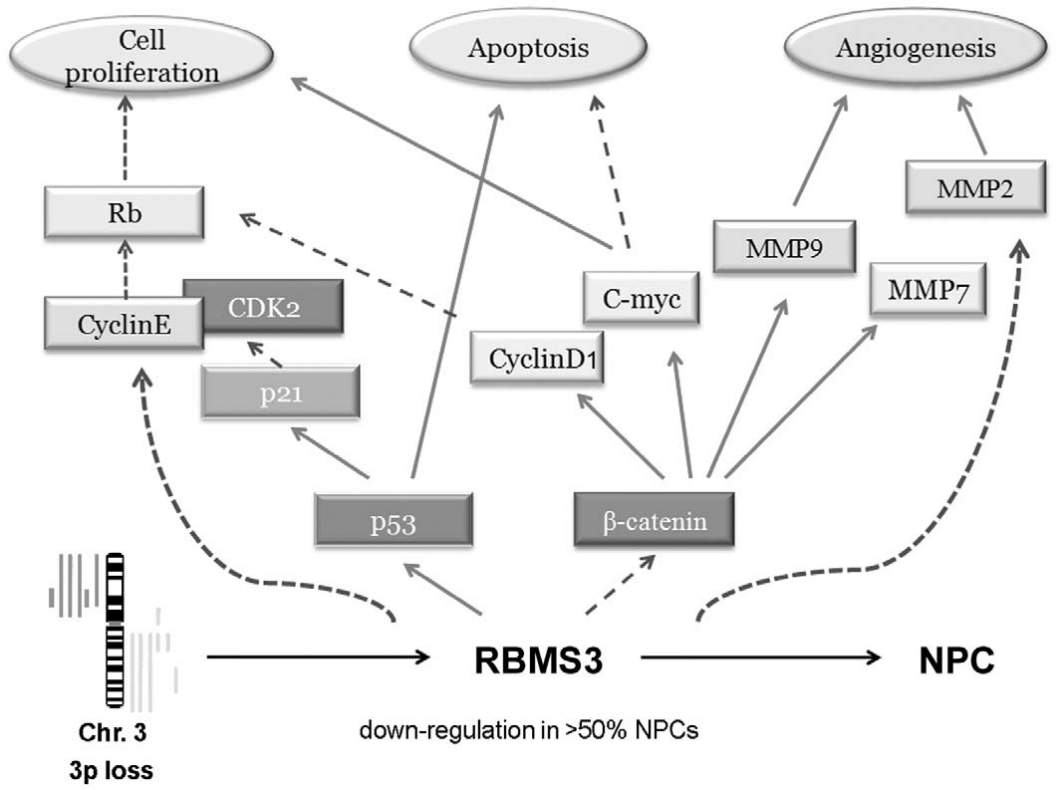
A schematic diagram showing the role of *RBMS3* in malignant transformation of nasopharyngeal carcinoma cell. Activation →, inhibition →.

Moreover, our group recently revealed that RBMS3 could directly bind to the promoter region of c-Myc [46], suggesting that RBMS3 also possesses the DNA binding activity. Based on our findings, we hypothesize that RBMS3 may regulate certain key proteins involved in cell cycle, apoptosis and angiogenesis. The potential molecular mechanisms underlying the RBMS3-mediated tumor suppression in NPC are summarized in Fig. 7. In conclusion, our data provide a foundation to explore the role of RBMS3 in the NPC pathogenesis. More comprehensive understanding of the tumor suppressive mechanisms of RBMS3 in NPC would provide much more effective therapeutic strategy for the management of NPC patients.

## Materials and Methods

### Ethics Statement

Tumor specimens used in this study were approved by the Committees for Ethical Review of Research involving Human Subjects at University of Hong Kong. Written Informed consent was obtained from the patients involved. Animal operation was carried out according to the protocols approved by the Committee on the Use of Live Animals in Teaching and Research (CULATRA). Lisence which could conduct live animal experiments was approved by Hong Kong Department of Health (lisence no. (09–770) in DH/HA&P/8/2/3 Pt.17).

### Cell Lines and Primary Tumor Specimens

EBV-positive NPC cell line C666 was derived from NPC tissue harbored latent EBV infection [47], [48], while EBV-negative NPC cell lines CNE2 and SUNE1 were derived from poorly differentiated NPC [49]. NP460 is an immortalized nasopharyngeal epithelium cell line [50]. Three NPC cell lines (C666, SUNE1, CNE2) and one immortalized nasopharyngeal epithelial cell lines (NP460) were kindly provided by Professor Tsao (Department of Anatomy, The University of Hong Kong). CNE2, SUNE1 and C666 were cultured in RPMI-1640 medium supplemented with 10% fetal bovine serum. NP460 cells were cultured in defined keratinocyte serum-free medium.

The primary NPC tumor tissues and their paired nontumorous tissues from 15 confirmed NPC patients (Age: 44–77 years; Female: n = 4; Male: n = 11) were collected immediately after surgical resection from the Queen Mary Hospital in Hong Kong. Samples used in this study were collected following methods approved by the Committee for Ethical Review of Research involving Human Subjects at the University of Hong Kong.

### Establishment of RBMS3 Expressing Cell Lines

The OmicsLink-RBMS3 was purchased from GeneCopoeia Inc. (Germantown, MD) and sub-cloned into pcDNA3.1 (+) (Invitrogen, Carlsbad, CA) according to the manufacture’s protocol. RBMS3 was then stably transfected into the NPC cell lines SUNE1 and CNE2 using LipofectamineTM 2000 (Invitrogen, Carlsbad, CA). Blank vector pcDNA3.1 (+) transfected cells (SUNE1-V1 and CNE2-V1) were used as controls.

### RNA Extraction, Reverse Transcription and PCR

Total RNA from cultured cells and tumor tissues were extracted using Trizol (Invitrogen, Carlsbad, CA) according to the manufacturer’s instructions. Reverse transcription of total RNA was performed using SuperScript III kit (Invitrogen, Carlsbad, CA). The expression of RBMS3 was determined by RT-PCR at RNA level using the appropriate primer set (RBMS3-F: 5′- ACAAGAGCAAGACCCAACA- AA-3′; RBMS3-R: 5′- TGTCCAAAGGGTTTCAGCATA-3′). Images were processed and analyzed with ImageJ software (Wayne Rashband, Nationlal Institute of Health, Bethesda, MD). Quantitative data were exported and *RBMS3* expression was normalized using *GAPDH* as a control.

### Quantitative Real-time PCR (qPCR)

qPCR was performed using the SYBR Green Supermix and the ABI7900HT Fast Real-Time PCR system (Applied Biosystems, Foster City, CA). RBMS3 specific primers (RBMS3-F: 5′- ACAAGAGCAAGACCCAACAAA-3′; RBMS3-R: 5′- TGTCCAAAGGGTTTCAGCATA-3′) were bought from GeneCopoeia Inc. (Germantown, MD). The 18s RNA was used as internal control. The assay was performed in triplicate and values were normalized using the internal control. PCR was performed for 40 cycles at 95°C for 10sec, 60°C for 20sec and 72°C for 15sec. PCR products were subjected to a dissociation curve analysis using the light cycler system to exclude amplification of nonspecific products. Quantitation of the PCR data was processed using the ΔC_T_ method as described previously [51].

### Tumor-suppressive Function of RBMS3

To test the tumor-suppressive function of *RBMS3,* stable *RBMS3*-expressing clones (SUNE1-R4, SUNE1-R5, CNE2-R1 and CNE2-R2) were selected for further study. Empty vector transfected NPC cells (SUNE1-V1 and CNE2-V1) were used as control. Cell proliferation assay and foci formation assay were carried out as described previously [13]. The *in vivo* tumor suppression ability of *RBMS3* was investigated using a tumor xenograft experiment. Approximately 2×10^6^ RBMS3-expressing cells and control cells were injected subcutaneously (s.c.) into the right and left hind legs of 4-week-old nude mice (n = 10 for each group), respectively. Tumor formation in nude mice was monitored over a 4-week period. The tumor volume was calculated by the formula V = 0.5×L×W^2^. Following sacrifice, tumors were excised, fixed in 10% formalin and embedded in paraffin block for IHC study.

### Small Interfering RNA Transfection

*RBMS3* expression was silenced by double-stranded siRNA of targeting of RBMS3 (sense 5′-CCAACCGCAUGAUUCCACAtt-3′; antisense 5′-UGUGGAAUCAUGC- GGUUGGtt-3′) and scramble siRNA, which were obtained from Ambion’s predesigned siRNA database (Ambion, Inc., Austin, TX). NP460 cells were transfected with siRNA using Lipofectamine™ 2000 (Invitrogen, Carlsbad, CA) according to the manufacture’s instruction. Forty-eight hours after transfection, the effect of gene-silencing was measured by qPCR and Western blot analysis. Scramble siRNA was used as a negative control.

### Cell Cycle Analysis

*RBMS3* and vector-transfected SUNE1 cells (1×10^6^) were cultured in RPMI medium containing 10% fetal bovine serum (FBS), while si-RBMS3 and si-scramble transfected NP460 cells were cultured in defined keratinocyte serum-free medium. Serum was withdrawn from culture medium when cells were 70% confluent. After 72 h, 10% FBS was added in the medium for an additional 12 h. Cells were fixed in 70% ethanol, stained with propidium iodide, and DNA content was analyzed using Cytomics FC (Beckman Coulter).

### Immunohistochemistry (IHC)

Tumor sections (5 µm thick) from primary NPCs or mouse xenografts were used for immunohistochemical analysis. Briefly, paraffin-embedded sections were deparaffinized, blocked with goat serum, followed by incubation with mouse anti- RBMS3 (1∶100) or anti-CD34 (1∶100) overnight at 4°C. After incubation with horseradish peroxidase linked secondary antibody for 30 min, the sections were counterstained with Mayer’s hematoxylin. For RBMS3 staining, the scores were determined by the intensity of staining: positive staining (moderate and strong nuclear staining) and downregulation (absent and weak staining).

### Microvessel Density Analysis

Tumor xenograft sections were stained for CD34, and four random medium-power fields (× 200) were photographed for each tumor. Vessels were counted by a blinded observer using standard criteria [52].

### Immunofluorescence (IF)

*RBMS3* and vector-transfected SUNE1 cells were grown on gelatin-coated cover slips, fixed with 4% paraformaldehyde, permeabilized in PBS, which contain 0.1% Triton-X 100, and blocked with 1% bovine serum albumin. The cells were then treated with antibodies targeting β-catenin (Cell Signaling Technology, Danvers, MA) at 4°C overnight. After washing with PBS, cells were incubated with FITC-conjugated anti-mouse secondary antibody and Texas red–conjugated anti-rabbit secondary antibody at room temperature for 1 hour. Antifade 4′, 6-diamidino-2-phenylindole (DAPI) solution was added, and images were captured.

### Transferase-mediated dUTP Nick-end Labeling (TUNEL) Assay

*RBMS3* and vector-transfected SUNE1 cells were treated with straurosporine (STS; 1 µM) for 2 hours. Morphological changes in the nuclear chromatin undergoing apoptosis were detected by terminal deoxynucleotidyl TUNEL assay according to the manufacturer’s protocol (Roche, Mannheim, Germany). Images were captured using a Leica DMRA fluorescence microscope (Rueil-Malmaison, France). Triplicate independent experiments were performed.

### Mitochondrial Membrane Potential Assay

A MitoPT™ JC-1 Detection kit (Immunochemistry Technologies, Bloomington, MN) was used to detect the loss of mitochondrial membrane potential (ΔΨ_m_). Briefly, cells were cultured on cover slips until 80% confluence was attained in a 6-well plate. Before and after STS treatment, cells were washed twice with phosphate-buffered saline (PBS) and incubated with the ΔΨ_m_-sensitive dyes JC-1 at 37°C for 15 min, and a fluorescence microscope was used to capture the image with a Leica DMRA.

### Cell Fractionation

Separation of nuclear and cytosolic fractions was performed using the NucBuster Protein Extraction kit (Novagen Inc, Madison, WI) according to the manufacturer’s instructions.

### Western Blot Analysis

Western blot was performed according to the standard protocol with antibodies for cyclin D1, β-actin, E-cadherin, MMP7, and MMP9 (Santa Cruz Biotechnology, Santa Cruz, CA); p21, p27, cyclin E, p53 caspase-8, and caspase-9, (Cell Signaling Technology, Danvers, MA); and RBMS3 (Abnova, Taiwan).

### Statistical Analysis

Statistical analysis was performed with the SPSS standard version 13.0. Results expressed as mean ± SD were analyzed using Student’s t test. Differences were considered significant when P value was <0.05.

## References

[1] Jeannel D, Bouvier G, Huber A (1999) Nasopharyngeal carcinoma: an epidemiological approach to carcinogenesis. Cancer Surv 33: 125–155.

[2] Parkin DM, Bray F, Ferlay J, Pisani P (2005) Global cancer statistics, 2002. CA Cancer J Clin 55: 74–108. 15761078 10.3322/canjclin.55.2.74

[3] Vokes EE, Liebowitz DN, Weichselbaum RR (1997) Nasopharyngeal carcinoma. Lancet 350: 1087–1091. 10213566 10.1016/S0140-6736(97)07269-3

[4] Yuan JM, Wang XL, Xiang YB, Gao YT, Ross RK, et al (2000) Preserved foods in relation to risk of nasopharyngeal carcinoma in Shanghai, China. Int J Cancer 85: 358–363. 10652427 10.1002/(sici)1097-0215(20000201)85:3<358::aid-ijc11>3.0.co;2-e

[5] Liebowitz D (1994) Nasopharyngeal carcinoma: the Epstein-Barr virus association. Semin Oncol 21: 376–381. 8209269

[6] Lo KW, Huang DP (2002) Genetic and epigenetic changes in nasopharyngeal carcinoma. Semin Cancer Biol 12: 451–462. 12450731 10.1016/s1044579x02000883

[7] Henle G, Henle W (1976) Epstein-Barr virus-specific IgA serum antibodies as an outstanding feature of nasopharyngeal carcinoma. Int J Cancer 17: 1–7. 175020 10.1002/ijc.2910170102

[8] Kwong D, Lam A, Guan X, Law S, Tai A, et al (2004) Chromosomal aberrations in esophageal squamous cell carcinoma among Chinese: gain of 12p predicts poor prognosis after surgery. Hum Pathol 35: 309–316. 15017586 10.1016/j.humpath.2003.10.020

[9] Hesson LB, Cooper WN, Latif F (2007) Evaluation of the 3p21.3 tumour-suppressor gene cluster. Oncogene 26: 7283–7301. 17533367 10.1038/sj.onc.1210547

[10] Qiu GH, Tan LK, Loh KS, Lim CY, Srivastava G, et al (2004) The candidate tumor suppressor gene BLU, located at the commonly deleted region 3p21.3, is an E2F-regulated, stress-responsive gene and inactivated by both epigenetic and genetic mechanisms in nasopharyngeal carcinoma. Oncogene 23: 4793–4806. 15122337 10.1038/sj.onc.1207632

[11] Qin YR, Fu L, Sham PC, Kwong DL, Zhu CL, et al (2008) Single-nucleotide polymorphism-mass array reveals commonly deleted regions at 3p22 and 3p14.2 associate with poor clinical outcome in esophageal squamous cell carcinoma. Int J Cancer 123: 826–830. 18508313 10.1002/ijc.23577

[12] Qiu GH, Salto-Tellez M, Ross JA, Yeo W, Cui Y, et al (2008) The tumor suppressor gene DLEC1 is frequently silenced by DNA methylation in hepatocellular carcinoma and induces G1 arrest in cell cycle. J Hepatol 48: 433–441. 18191269 10.1016/j.jhep.2007.11.015

[13] Fu L, Qin YR, Xie D, Hu L, Kwong DL, et al (2007) Characterization of a novel tumor-suppressor gene PLC delta 1 at 3p22 in esophageal squamous cell carcinoma. Cancer Res 67: 10720–10726. 18006814 10.1158/0008-5472.CAN-07-2411

[14] Hu XT, Zhang FB, Fan YC, Shu XS, Wong AH, et al (2009) Phospholipase C delta 1 is a novel 3p22.3 tumor suppressor involved in cytoskeleton organization, with its epigenetic silencing correlated with high-stage gastric cancer. Oncogene 28: 2466–2475. 19448674 10.1038/onc.2009.92

[15] Penkov D, Ni R, Else C, Pinol-Roma S, Ramirez F, et al (2000) Cloning of a human gene closely related to the genes coding for the c-myc single-strand binding proteins. Gene 243: 27–36. 10675610 10.1016/s0378-1119(99)00515-6

[16] Arvanitis C, Felsher DW (2006) Conditional transgenic models define how MYC initiates and maintains tumorigenesis. Semin Cancer Biol 16: 313–317. 16935001 10.1016/j.semcancer.2006.07.012

[17] Fan CS, Wong N, Leung SF, To KF, Lo KW, et al (2000) Frequent c-myc and Int-2 overrepresentations in nasopharyngeal carcinoma. Hum Pathol 31: 169–178. 10685630 10.1016/s0046-8177(00)80216-6

[18] Hui AB, Lo KW, Teo PM, To KF, Huang DP (2002) Genome wide detection of oncogene amplifications in nasopharyngeal carcinoma by array based comparative genomic hybridization. Int J Oncol 20: 467–473. 11836556

[19] Fritz D, Stefanovic B (2007) RNA-binding protein RBMS3 is expressed in activated hepatic stellate cells and liver fibrosis and increases expression of transcription factor Prx1. J Mol Biol 371: 585–595. 17586524 10.1016/j.jmb.2007.06.006 PMC1976254

[20] Wang XH, Sun X, Meng XW, Lv ZW, Du YJ, et al (2010) beta-catenin siRNA regulation of apoptosis- and angiogenesis-related gene expression in hepatocellular carcinoma cells: potential uses for gene therapy. Oncol Rep 24: 1093–1099. 20811694

[21] Carnero A, Hannon GJ (1998) The INK4 family of CDK inhibitors. Curr Top Microbiol Immunol 227: 43–55. 9479825 10.1007/978-3-642-71941-7_3

[22] Lu Z, Ghosh S, Wang Z, Hunter T (2003) Downregulation of caveolin-1 function by EGF leads to the loss of E-cadherin, increased transcriptional activity of beta-catenin, and enhanced tumor cell invasion. Cancer Cell 4: 499–515. 14706341 10.1016/s1535-6108(03)00304-0

[23] Sherr CJ, Roberts JM (1999) CDK inhibitors: positive and negative regulators of G1-phase progression. Genes Dev 13: 1501–1512. 10385618 10.1101/gad.13.12.1501

[24] Hoshino I, Matsubara H, Akutsu Y, Nishimori T, Yoneyama Y, et al (2007) Tumor suppressor Prdx1 is a prognostic factor in esophageal squamous cell carcinoma patients. Oncol Rep 18: 867–871. 17786348

[25] Giorgio M, Trinei M, Migliaccio E, Pelicci PG (2007) Hydrogen peroxide: a metabolic by-product or a common mediator of ageing signals? Nat Rev Mol Cell Biol 8: 722–728. 17700625 10.1038/nrm2240

[26] Pellegrini M, Pacini S, Baldari CT (2005) p66SHC: the apoptotic side of Shc proteins. Apoptosis 10: 13–18. 15711918 10.1007/s10495-005-6057-8

[27] Petronilli V, Costantini P, Scorrano L, Colonna R, Passamonti S, et al (1994) The voltage sensor of the mitochondrial permeability transition pore is tuned by the oxidation-reduction state of vicinal thiols. Increase of the gating potential by oxidants and its reversal by reducing agents. J Biol Chem 269: 16638–16642. 7515881

[28] Bernardi P, Petronilli V, Di Lisa F, Forte M (2001) A mitochondrial perspective on cell death. Trends Biochem Sci 26: 112–117. 11166569 10.1016/s0968-0004(00)01745-x

[29] Bouck N, Stellmach V, Hsu SC (1996) How tumors become angiogenic. In: Advances in Cancer Research, Vol VandeWoude GFKG, editor. 69: 135–174. 10.1016/s0065-230x(08)60862-3 8791681

[30] Mukhopadhyay D, Tsiokas L, Sukhatme VP (1995) Wild-type p53 and v-Src exert opposing influences on human vascular endothelial growth factor gene expression. Cancer Res 55: 6161–6165. 8521408

[31] Rak J, Mitsuhashi Y, Bayko L, Filmus J, Shirasawa S, et al (1995) Mutant ras oncogenes upregulate VEGF/VPF expression: implications for induction and inhibition of tumor angiogenesis. Cancer Res 55: 4575–4580. 7553632

[32] Kraemer M, Tournaire R, Dejong V, Montreau N, Briane D, et al (1999) Rat embryo fibroblasts transformed by c-Jun display highly metastatic and angiogenic activities in vivo and deregulate gene expression of both angiogenic and antiangiogenic factors. Cell Growth Differ 10: 193–200. 10099833

[33] Marconcini L, Marchio S, Morbidelli L, Cartocci E, Albini A, et al (1999) c-fos-induced growth factor/vascular endothelial growth factor D induces angiogenesis in vivo and in vitro. Proc Natl Acad Sci U S A 96: 9671–9676. 10449752 10.1073/pnas.96.17.9671 PMC22268

[34] Gnarra JR, Zhou S, Merrill MJ, Wagner JR, Krumm A, et al (1996) Post-transcriptional regulation of vascular endothelial growth factor mRNA by the product of the VHL tumor suppressor gene. Proc Natl Acad Sci U S A 93: 10589–10594. 8855222 10.1073/pnas.93.20.10589 PMC38197

[35] Claudio PP, Stiegler P, Howard CM, Bellan C, Minimo C, et al (2001) RB2/p130 gene-enhanced expression down-regulates vascular endothelial growth factor expression and inhibits angiogenesis in vivo. Cancer Res 61: 462–468. 11212232

[36] Ahn GO, Brown JM (2008) Matrix metalloproteinase-9 is required for tumor vasculogenesis but not for angiogenesis: role of bone marrow-derived myelomonocytic cells. Cancer Cell 13: 193–205. 18328424 10.1016/j.ccr.2007.11.032 PMC2967441

[37] van Noort M, Meeldijk J, van der Zee R, Destree O, Clevers H (2002) Wnt signaling controls the phosphorylation status of beta-catenin. J Biol Chem 277: 17901–17905. 11834740 10.1074/jbc.M111635200

[38] Iwai S, Yonekawa A, Harada C, Hamada M, Katagiri W, et al (2010) Involvement of the Wnt-beta-catenin pathway in invasion and migration of oral squamous carcinoma cells. Int J Oncol 37: 1095–1103. 20878057 10.3892/ijo_00000761

[39] Taurin S, Sandbo N, Qin Y, Browning D, Dulin NO (2006) Phosphorylation of beta-catenin by cyclic AMP-dependent protein kinase. J Biol Chem 281: 9971–9976. 16476742 10.1074/jbc.M508778200

[40] Morrison JA, Gulley ML, Pathmanathan R, Raab-Traub N (2004) Differential signaling pathways are activated in the Epstein-Barr virus-associated malignancies nasopharyngeal carcinoma and Hodgkin lymphoma. Cancer Res 64: 5251–5260. 15289331 10.1158/0008-5472.CAN-04-0538

[41] Zeng ZY, Zhou YH, Zhang WL, Xiong W, Fan SQ, et al (2007) Gene expression profiling of nasopharyngeal carcinoma reveals the abnormally regulated Wnt signaling pathway. Hum Pathol 38: 120–133. 16996564 10.1016/j.humpath.2006.06.023

[42] Luo J, Xiao J, Tao Z, Li X (1997) Detection of c-myc gene expression in nasopharyngeal carcinoma by nonradioactive in situ hybridization and immunohistochemistry. Chin Med J (Engl) 110: 229–232. 9594347

[43] Porter MJ, Field JK, Leung SF, Lo D, Lee JC, et al (1994) The detection of the c-myc and ras oncogenes in nasopharyngeal carcinoma by immunohistochemistry. Acta Otolaryngol 114: 105–109. 8128845 10.3109/00016489409126025

[44] Fang YJ, Lu ZH, Wang GQ, Pan ZZ, Zhou ZW, et al (2009) Elevated expressions of MMP7, TROP2, and survivin are associated with survival, disease recurrence, and liver metastasis of colon cancer. Int J Colorectal Dis 24: 875–884. 19421758 10.1007/s00384-009-0725-z

[45] Yue W, Sun Q, Landreneau R, Wu C, Siegfried JM, et al (2009) Fibulin-5 suppresses lung cancer invasion by inhibiting matrix metalloproteinase-7 expression. Cancer Res 69: 6339–6346. 19584278 10.1158/0008-5472.CAN-09-0398 PMC2719681

[46] Li Y, Chen L, Nie CJ, Zeng TT, Liu H, et al (2011) Downregulation of RBMS3 is associated with poor prognosis in esophageal squamous cell carcinoma. Cancer Res 71: 6106–6115. 21844183 10.1158/0008-5472.CAN-10-4291

[47] Hui AB, Cheung ST, Fong Y, Lo KW, Huang DP (1998) Characterization of a new EBV-associated nasopharyngeal carcinoma cell line. Cancer Genet Cytogenet 101: 83–88. 9494607 10.1016/s0165-4608(97)00231-8

[48] Cheung ST, Huang DP, Hui AB, Lo KW, Ko CW, et al (1999) Nasopharyngeal carcinoma cell line (C666–1) consistently harbouring Epstein-Barr virus. Int J Cancer 83: 121–126. 10449618 10.1002/(sici)1097-0215(19990924)83:1<121::aid-ijc21>3.0.co;2-f

[49] Sizhong Z, Xiukung G, Yi Z (1983) Cytogenetic studies on an epithelial cell line derived from poorly differentiated nasopharyngeal carcinoma. Int J Cancer 31: 587–590. 6852976 10.1002/ijc.2910310509

[50] Li HM, Man C, Jin Y, Deng W, Yip YL, et al (2006) Molecular and cytogenetic changes involved in the immortalization of nasopharyngeal epithelial cells by telomerase. Int J Cancer 119: 1567–1576. 16688717 10.1002/ijc.22032

[51] Livak KJ, Schmittgen TD (2001) Analysis of relative gene expression data using real-time quantitative PCR and the 2(T)(-Delta Delta C) method. Methods 25: 402–408. 11846609 10.1006/meth.2001.1262

[52] Fox SB (2001) Microscopic assessment of angiogenesis in tumors. Methods Mol Med 46: 29–46. 21340909 10.1385/1-59259-143-4:029

